# World Peace in One Hour Peace is Profitable Roadmap to Peace

**DOI:** 10.7189/jogh.08-010205

**Published:** 2018-06

**Authors:** Nenad Bach

*You may say I’m a dreamer*
*But I’m not the only one*
*I hope some day you’ll join us*
*And the world will be as one*
from *Imagine* by John W. Lennon

Is this just a song or wishful thinking?

Are these just words or is it prophecy?

Is humanity capable of achieving sustainable, everlasting peace?

I say YES and the time is NOW! This is a roadmap, a step-by-step manual.

## WE HAVE EVERYTHING WE NEED TO ACHIEVE WORLD PEACE IN ONE HOUR

### FACT 1

Twitter goes around the world in less than a second. Technology is on our side. We can inform more than a billion people in a single day. Fact! The number of smartphones and digital cameras is in the ballpark of 3 billion. Violence cannot be hidden any more.

**Figure:**
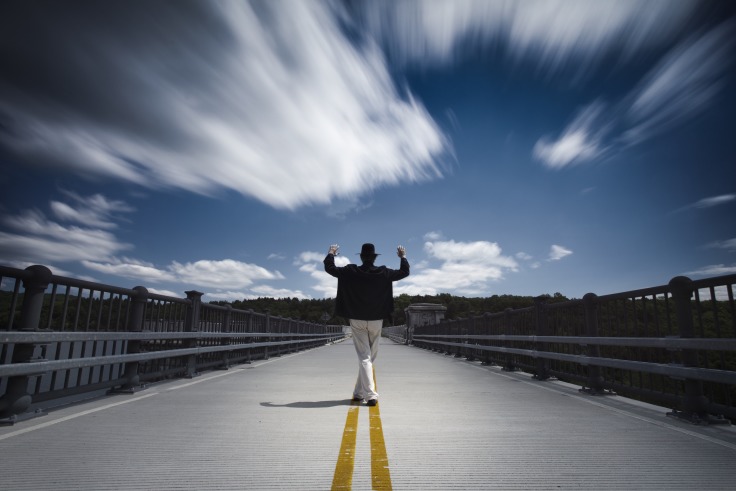
Photo by Ilija Veselica

### FACT 2

The Western Hemisphere has been a War-Free zone since April 17th 2017. A year ago, most of us couldn’t have even IMAGINEd this happening. Fact!

So why do wars happen? What is the single most common denominator in all the wars throughout known human history? The answer is easy to find - just follow the money.

Where there’s Justice, Peace follows.

Where there’s Balance, Peace follows.

Where there’s Love, Peace follows.

### FACT 3

How many people have died in all the wars, massacres, slaughters and oppressions of the 20^th^ century? A few atrocitologists like Mahmoud Cherif Bassiouni (died on Sept 25^th^ 2017) and Zbigniew Brezinski (died on May 26^th^ 2017), have made estimates; depending on the source, between 167 and 203 million humans have been killed in the 20^th^ century – roughly 2 million per year. In this, 21^st^ century, it has so far been 10-20 times less. Fact! Interestingly enough, both atrocitologists died in 2017, a few months apart, sending us a poetic message: “We gave you the numbers, now it’s up to you people.”

### FACT 4

Global military expenditure officially stands at over US$ 1.7 trillion in annual expenditure. IMAGINE what could be done with 1 trillion every year to benefit humanity.

### FACT 5

According to the Stockholm International Peace Research Institute: The volume of international major weapons transfers were 8.4% higher between 2012-16 than between 2007-11. This was the highest volume for any five-year period since 1990. The four biggest exporters in 2012-16 were the United States, Russia, China and France, by rank. The four biggest importers were India, Saudi Arabia, the United Arab Emirates (UAE) and China. The flow of arms to Asia and Oceania and the Middle East increased between 2007-11 and 2012-16, while there was a decrease in the flow to Europe, the Americas and Africa.

The top 4 exporters of the conventional weapons – The United States, Russia, China and France – make a staggering total of close to 70% of all arms trade.

### FACT 6

The same 4 countries are the United Nations Permanent Security Council members (source: http://www.un.org/en/sc/members/).

### FACT 7

There are an estimated 110 million active landmines in the world and an equal amount of stockpile to be planted or destroyed. According to MineSweepers Towards a Landmine-Free World (http://www.landminefree.org/207/index.php/support/facts-about-landmines), war residuals kill or maim 5000 people per year, with over 40% being children. While the number of animals remains unaccounted for, the Animal Casualties of Underground War report by Adam M. Roberts and Kevin Stewart gives us an indication; In Sri Lanka, as many as 20 Asian elephants are killed by mines every year, according to zoologist Charles Santiapillai of the University of Peradeniya. Thousands of miles away, in Africa, land mines have ravaged wildlife, including threatened and highly endangered species. Mines have reportedly killed more than 100 elephants in Mozambique. Scott Nathanson, a Disarmament Campaign organizer, writes that elephants in the Gorongosa national game park “have been maimed because of anti-personnel land mines, or killed because of anti-tank mines.”

In Croatia, Professor Djuro Huber of the University of Zagreb has also documented wildlife fatalities due to landmines. His reports note the deaths of European brown bears, roe, deer, lynxes, and foxes as a result of mines placed in the region from 1990 to 1996.

It costs between US$ 3-30 to produce a landmine; To disable one, between US$ 300-1000. What is the price of pollution, of the inability to use agricultural land? What is the cost of losing a whole species? What is the actual cost of war?

## INTERLUDE

Each and every empire perished due to one common trait... arrogance of power. Every dictatorship fell because arrogance is not sustainable. It is an unnatural state of mind. On the contrary, PEACE is sustainable and cumulative.

Those who make profit in wars, do not care how they make the profit, as long as they do, so let’s keep that in mind in the final equation-formula for World Peace in One Hour.

After all is said and done, what is left after our personal existence and known civilization is Art, Architecture, Music, Poetry, Literature, Film, Philosophy... so let’s then start with the art.

### ACT 1

**Creation of the musical album World Peace in One Hour** in Lancaster, Pennsylvania (or place TBA) with at least one song played on all the instruments of the world or as many as we can find. On April 14^th^ 2018 we will jumpstart the project with, hopefully, a celebration of one year of peace in the western hemisphere (http://www.artsmu.com/event/019927de2216324a5cb2ad44c0902b0e). Establish a Facebook page World Peace in One Hour, where anybody can submit the World Peace in One Hour Sign. Wherever I go and salute, people, spontaneously, do the same with a smile. An example in Beijing, China below.

**Figure:**
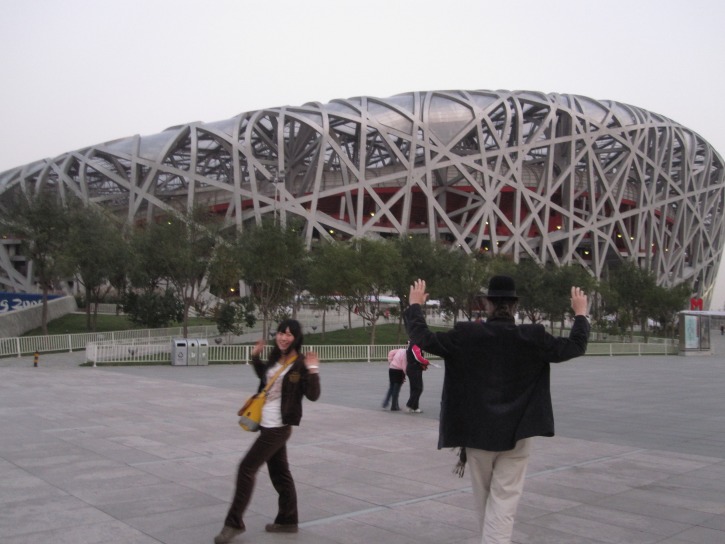
Photo by Vjera Bach

Once 2% of the humans think it is possible, it will happen.

On August 3^rd^ 2018 we’ll open an art exhibition at the Cercantico Gallery in Northern Italy on Lago di Garda with the photos of the World Peace in One Hour sign.

*Too many leaders, no one to follow*
*Too many clouds, not enough rain*
*Too many words and promises too hard to swallow*
*Too many innocents died in vain*
from I Will Follow You, by Nenad N. Bach

### ACT 2

**Peace is not just profitable but inevitable.** A pre-peace treaty signed by the G8 countries, plus as many other countries as possible, hopefully all of them, on June 21^st^ 2019 in Dubrovnik, (or place TBA) Croatia, a city/state known for its early democracy. So what will the treaty be about? Our proposal, suggestion, is to keep the military defense budgets as they are but immediately transform 50% of the arms production into the production of green energy and sustainable global infrastructure connecting continents with tunnels and vactrains; transforming cities with bullet trains instead of bullets. Don’t know how? Ask Elon Musk. For example, a landmine producer with 500 employees will stop producing landmines and start producing wind turbines. The government is the biggest employer in every country and therefore capable of transitioning in a timely fashion - quickly. For another example, if the government orders electric cars for its operation, it will speed up the process of transformation from fossil fuels to sustainable energy. Use part of that budget for research in medicine, global health, innovations in technology and humanity, caring about the environment, ecological sustainability, employing former soldiers, and giving them a new and self-rewarding purpose.

### ACT 3

**Signing a permanent World Peace at the UN Headquarters in New York on June 21^st^ 2020** at 11 am As the music album World Peace in One Hour (60 minutes in length) plays, all 193 UN members sign the treaty. All over the world, 60-minute concerts are held at the same time. At 12 noon, World Peace is proclaimed.

Now, rightfully so, you ask, what if some country or a madman dictator decide to unilaterally start a war? My answer: Nobody starts a war to lose. Therefore, if 192 countries jump on that one, any attempt to disrupt the peace would last 6-12 hours. Furthermore, we have to work on a self-correcting mechanism that will not allow violence. We have to use the power of technology.

The recent critical mass of sexual harassment cases didn’t happen because predators got enlightened, but because they couldn’t get away with it anymore, as they used to. The same critical mass will happen in regards to war.

So don’t be afraid to turn your Selfie into Self-Defense, as a selfless instrument of humanity. Report injustice and reward nobility; Stop the violence and support the kindness, just by using your Smartphone as an active witness for the common good. We have passed the Stone Age and the Bronze Age. As we are entering the Digital Age, The Age of the common woman and man, don’t wait – Participate!

Someone might say that armies will become obsolete. On the contrary, armies will protect The Peace, but soldiers will never again be in a position to harm or violate the innocent.

## IN CONCLUSION

War is profitable for 500 people and miserable for the rest of us.

Peace is profitable for all of us, including those 500.

An important benefit of this idea is that the Power Structure (Ratio) stays the same, because all countries redirect 50% of their defense budget towards the already-described domains, therefore, no country becomes more threatening or threatened. On the contrary, each country becomes less threatening and less threatened because their citizens are more satisfied and feel more unified with their respective governments. So the Power Structure stays the same, therefore, we as a civilization focus more on construction rather than destruction, finally realizing that HEAVEN IS HERE.

We, as the species that run the planet Earth, are at a crossroads of either self-destruction or an unimaginably progressive future as the Species of the Universe. Now is the time to make the right decision and focus our knowledge, resources, and talent towards the improvement of our lives on Mother Earth. Culture, Global Health, Education, Art, Science, Infrastructure, Environment, Music, Freedom of Expression, Beauty, and Wisdom will follow where there is no violence. Can you imagine unforeseen benefits?

Can we go higher? We can do it. We are better than this.

**World Peace in One Hour**

Nenad N. Bach

Written at The Black Cow on January 1^st^ 2018, Croton on Hudson, New York, USA

p.s. If you believe this is possible, sign the link below and spread the good news in your neighborhood, village, city, country or continent. We will inform you, and you will inform us about the progress.

p.p.s. If you play any of the 500+ known musical instruments, and you desire to be part of the World Peace in One Hour Orchestra’s attempt to qualify for The Guinness Book of Records, sign the link below.

p.p.p.s. If you sing and want to be a voice in the World Peace in One Hour Choir’s attempt to qualify for The Guinness Book of Records, sign the link below.

petition.worldpeaceinonehour.com

